# Qualitative evaluation of My Life Today - A co-produced personal tool from the IDEAL programme to help people with dementia monitor valued aspects of their lives

**DOI:** 10.1177/14713012241306506

**Published:** 2024-12-12

**Authors:** Claire Pentecost, Anna Hunt, Rachael Litherland, Catherine Quinn, Catherine Charlwood, Robin G Morris, Linda Clare

**Affiliations:** University of Exeter Medical School, 3286 University of Exeter, UK; University of Exeter Medical School, 3286 University of Exeter, UK; Innovations in Dementia CIC, UK; Centre for Applied Dementia Studies, 1905 University of Bradford, Bradford, UK; Wolfson Centre for Applied Health Research, UK; University of Exeter Medical School, 3286 University of Exeter, UK; Department of Psychology, King’s College London, Institute of Psychiatry, Psychology and Neuroscience, London, UK; University of Exeter Medical School, 3286 University of Exeter, UK; NIHR Applied Research Collaboration South-West Peninsula, UK

**Keywords:** Alzheimer’s, self-management, qualitative, realist, co-production

## Abstract

**Background and objectives:** Self-management of chronic conditions can help individuals take control of their health, both improving well-being and reducing the burden on health and social care resources. This study explored the potential of our co-produced self-management tool, My Life Today (MLT), to help people with dementia identify, plan and monitor aspects of their lives that are important to them and that help to maintain or improve well-being.

**Research design and methods:** We asked people with dementia to try using MLT. We conducted semi-structured interviews after one month and further interviews one month later. We also interviewed people who had supported the person with dementia to use MLT (‘supporters’). We adopted a realist approach to thematic analysis to explore what works, for whom, under what circumstances.

**Results:** Sixteen people with dementia and four supporters took part. All but one had mild to moderate cognitive impairment, and one had severe cognitive impairment. People with dementia used MLT according to their perceptions of its usefulness, their capabilities, and whether they had support. Using MLT helped most to think more positively about their activities and achievements and feel reassured by identifying the activities they were doing. Supporters and some people with dementia also derived benefits from planning and problem-solving to include more pleasurable activities.

**Discussion and implications:** People with dementia and supporters found MLT a helpful tool. Mechanisms of engagement with MLT resonate with theories of behaviour change concerning the evaluation of capabilities and feelings of confidence in the ability to complete MLT and plan activities. Offering simple self-management tools such as MLT could form part of a post-diagnostic support package for people with dementia. Providing flexibility in when tools are offered and how they are used can allow for differences in attitudes and capabilities and increase the likelihood of engagement.

## Introduction

Among the growing population of people with dementia in the UK, around two-thirds live at home (Alzheimer’s Society, 2020) and around 85% wish to stay there for as long as possible (Alzheimer’s Society, 2016). In this context, better supporting self-management of a person’s condition, as emphasised by the UK National Health Service (NHS) ‘Long Term Plan’ (NHS, 2019), can help people with dementia to live well at home for longer, and can reduce the need for health and social care services. According to Lorig and Holman (2003), self-management in health care involves increasing a patient’s power and responsibility for making decisions and ensuring necessary health care actions are taken and involves three tasks: 1. Medical management such as medication, diet, and exercise, 2. Maintaining, changing and creating new meaningful behaviours, and 3. Dealing with emotional sequala. For people with mild-to-moderate dementia, self-management should be interpreted as managing life with dementia rather than the dementia itself (Martin et al., 2015), and encouraging a more empowered approach, as this can enhance self-efficacy (Quinn et al., 2022). Studies have demonstrated that people with mild dementia can engage in and benefit from supported self-management involving identifying, setting and working on personalised goals (Quinn et al. 2016b; Jennings et al., 2018; Clare et al., 2023). Despite this, initiatives encouraging self-management in health settings rarely include people with dementia.

Self-management should address the patients’ perceived problems (Lorig & Holman, 2003). One of the challenges highlighted by people with dementia is finding ways of continuing to independently engage in meaningful and enjoyable activities. People with dementia can nevertheless find ways to minimise the impacts of the condition and continue with, adapt, or find alternatives to such activities (Pentecost et al., 2022). This can occur at a psychological level by developing coping responses to the progression of dementia (Bjørkløf et al., 2019), helping to enhance feelings of identity and well-being (Stapley et al., 2023) and countering depression (Blomberg et al., 2024). Engagement in social interactions and maintenance of close personal relationships are especially important for maintaining well-being (Austin et al., 2016; Martyr et al., 2018). Any behaviours that enhance or maintain connections with individuals or the wider community, attachment to immediate surroundings, physical health, or a sense of purpose, can contribute to enhancing well-being for people with dementia (O’Rourke et al., 2015).

Previously, a 57-item inventory was designed for caregivers and providers to complete on behalf of or with a person with dementia to discover and target potentially pleasant activities that could reduce depression (Teri & Logsdon, 1991). A list of 57 specific activities such as ‘recalling and discussing past events’ and ‘looking at photo albums’ are rated according to enjoyability and then used as a priority list. However, as far as we are aware no self-management tools have been created by and for people with dementia to use that can help them identify and target the things that are personally important to them in maintaining meaningful behaviours that impact quality of life.

Involving people with lived experience of dementia in co-producing self-management resources can increase the likelihood that they will be fit-for-purpose. Consequently, as a core aim throughout the IDEAL (Clare et al., 2014; Silarova et al., 2018) programme, we sought to work with people with dementia to co-produce new tools to support well-being and quality of life for people with dementia, for example, the Living with Dementia Toolkit and the My Life Questionnaire (Clare et al., 2023). Based on our findings emphasising the broad range of influences on quality of life (Clare et al., 2019; Martyr et al., 2018), and on discussions with our involvement group, known as the ALWAYs group (Litherland et al., 2018), we also aimed to develop a tool that could help people with dementia to continue engaging with aspects of their lives that are meaningful. The new tool is called My Life Today (MLT). The qualitative study reported here was designed to evaluate the usefulness of MLT for people with dementia and identify any further improvements that could be incorporated into a future version to enhance its usefulness. We set out to answer the questions: Is MLT helpful to identify valued aspects of participants’ daily lives, to monitor these things, and assist with planning future activities? If so, how is it useful, who is it useful for, and under what circumstances?

## Methods

The COREQ checklist (Tong et al., 2007) has been used as a reporting framework for this study and a copy of the completed checklist can be found in the Supplementary file.

### The My Life Today resource

We worked with a co-production group consisting of six people with dementia. The aim was to produce a tool to allow individuals with mild-to-moderate dementia to identify and monitor the activities, events, and experiences in their daily lives that help to maintain or improve well-being and quality of life, acknowledging that these will be different for each person. The co-production group worked with the research team in several online meetings during 2022 to agree the content and design of the tool and prepare a draft with accompanying information and illustrative examples. Members of the co-production group tested a draft version between meetings and fed back suggestions for improvements. We made changes and shared and discussed the first version of the tool with a stakeholder group of clinicians and researchers in the dementia field, who were asked ‘Who will it be useful for?’, ‘How might it be helpful?’ and ‘Why might it be helpful’ based on realist principles (Pawson & Tilley, 1997). These responses were used to help develop initial theories to test in the evaluation. They were also asked for any suggestions for improvements and these minor design changes were incorporated, resulting in the second version, the My Life Today (MLT) booklet tested in this study. We also created a short explanatory video demonstrating how to use the booklet. The MLT booklet contains information about its purpose, instructions, and tips to stay motivated to use it. The person with dementia completing the booklet is asked to write answers to questions in a chart. These questions are: 1. What makes me happy or helps me feel good? 2. How often do I want to do this thing (that makes me happy or helps me feel good)? 3. Am I doing this thing often enough? Yes or no. 4. If you said ‘no’, think about what would help you do it more often? 5. What action could I take to make this thing happen? The chart allows for entries over several weeks and there is an area for notes on each page to reflect on progress.

### Ethics

The IDEAL programme operates under NHS approval from Wales 5 REC (reference 18/SS/0037). Approval for the MLT study was granted by the University of Exeter Medical School & Health and Care Professionals Research Ethics Committee (reference 529548) on 26/01/2023. All participants provided verbal recorded informed consent.

#### Participants and procedure

Participants were identified through Join Dementia Research (https://www.joindementiaresearch.nihr.ac.uk/). We also invited expressions of interest via the DEEP website (https://www.dementiavoices.org.uk/deep-news/deep-updates/) and newsletter. Inclusion criteria were a self-reported diagnosis of dementia and the ability to communicate verbally in English by telephone or Zoom.

##### Consent

All potential participants were contacted by telephone or email and provided with verbal information and/or written information sheets. After having time to consider taking part, informed consent was obtained and then demographic information was collected. To profile dementia severity, for those willing to be assessed, the 5-min telephone Montreal Cognitive Assessment (MoCA) (Wong et al., 2015) was administered. Participants were instructed how to watch the MLT eight-minute online explanatory video, either via a QR code in the booklet or via a link sent by email. MLT was then posted to participants and researchers arranged a telephone call one week later to answer any further questions. A follow-up interview was then arranged after around four weeks.

#### Interviews

Experienced dementia researchers (CP, AH) were allocated half of the participants each and used a topic guide to structure conversational style interviews (Appendix 1) either by telephone or via Zoom as preferred. The questions explored the understanding of the purpose of MLT, how it was completed, whether it was useful and why, any help received, and any comments on the design and content. As the work progressed researchers also asked questions based on ‘what works, for whom, in what circumstances’ theories derived from interviews with other participants and ongoing analysis. After each interview, the researchers made notes of the main points and possible follow-up questions to ask in a second interview. The two researchers shared their notes. At the end of the interview, people with dementia were asked if they would like to continue to use MLT and if so, another interview was arranged to see whether, how, and why their use of MLT had changed. They were also asked if anyone had helped them to use MLT, and whether or not they wanted to nominate that person to be interviewed. All nominated ‘supporters’ were approached and given information, and if willing, provided verbal informed consent to participate in an interview (Appendix 1) to answer questions about the support they provided, their impressions of usefulness for the person with dementia, and design suggestions. All interviews were audio recorded.

#### Analysis

Following the principles of realist evaluation (Pawson & Tilley, 1997) we applied inductive and deductive logic and the emerging understanding of the data to theorise why the observed outcomes from using MLT occurred drawing from realist interpretations of thematic analysis (Kiger & Varpio, 2020; Wiltshire & Ronkainen, 2021). This involved following the common stages of thematic analysis (Braun & Clarke, 2006): familiarisation, generating initial codes, searching for themes, reviewing themes, defining themes and checking fit with the data, and presenting the data whilst attempting to explain why MLT may be useful for whom, why and in what circumstances (Greenhalgh et al., 2017).

Transcribed interviews were uploaded to and analysed within NVivo14 version 14.23.1 (Lumivero, 2023). Both researchers familiarised themselves with the data by reading and re-reading the interviews and interview notes. Any thoughts, impressions, or ideas generated during familiarisation were added to the notes for each participant. Using a collaborative analysis approach to seek accuracy, reliability, and consensus (Wiltshire & Ronkainen, 2021) the researchers (CP and AH) then coded the first completed interview independently. Any text indicating contextual factors (elements of the backdrop of presenting MLT to people having an impact on the outcome), mechanisms (the way people responded to MLT) or outcomes (intended or unintended observable effects based on context and mechanism interactions), and any other potentially relevant observations were coded. Researchers then compared and agreed on the coding and code descriptions for that interview. Coding for the remaining interviews continued independently as new interviews were collected and coded. New codes were discussed and agreed upon, and existing codes were refined iteratively via discussion. Alongside coding, initial theories about ‘what works, for whom, under what circumstances’ were jointly developed and tested in new interviews and against existing extracted coded data in charts. Once all interviews were coded, analysts reworked the theories to test plausible inferences against the whole dataset based on retroductive reasoning and plausible implications for a wider sample (Wiltshire & Ronkainen, 2021).

## Findings

We approached 76 potential participants, 31 did not respond, three had passed away, 11 were interested but unable to participate due to illness, and 12 were not interested. We recruited 19 people with dementia. Three withdrew before the first interview; one was caring for a partner with severe dementia, and two had become unwell. This left 16 participants with dementia, of whom seven participated in a second interview. Four of the 16 nominated a supporter who had assisted them in using My Life Today and these supporters were interviewed after the last interview with the person with dementia; three were spouses and one was a care worker.

### Sample characteristics

Demographic characteristics of participants can be found in Table 1 and demographic characteristics of supporters and their relationship to the person with dementia can be found in Table 2.

**Table 1.** Demographic characteristics - people with dementia.

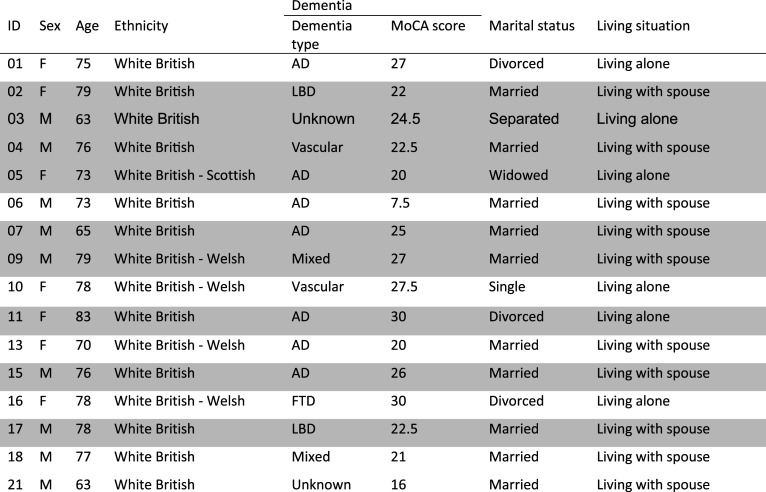

^J^ Pt 02 2^nd^ interviews were joint with their nominated supporter; Shading indicates 2 interviews undertaken; AD - Alzheimer’s disease; LBD - Lewy body dementia; Mixed - Alzheimer’s disease / vascular dementia; FTD - Frontotemporal dementia; MoCA score range is 0–30, 10 or less indicates severe cognitive impairment, 11-18 moderate cognitive impairment, 19-25 mild cognitive impairment and 26-30 normal cognition (Fasnacht et al., 2023).

**Table 2.** Demographic characteristics – supporters.

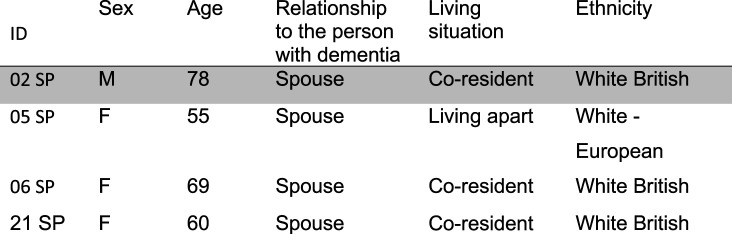

Shading indicates 2 interviews undertaken, 02SP jointly with 02.

All participants were willing to complete the 5-min telephone Montreal Cognitive Assessment (Wong et al., 2015). The mean MoCA score for people with dementia was 23 (range 7.5–30). The MoCA score range is 0–30. A score of 0–10 indicates severe cognitive impairment, 11-18 moderate cognitive impairment, 19-25 mild cognitive impairment, and 26-30 normal cognition (Fasnacht et al., 2023). Only one person had a score indicating severe cognitive impairment. The mean MoCA score for those who participated in a second interview was 23.6 compared with 22.6 for those who did not. The most common dementia type was Alzheimer’s disease (*n* = 7) and one or two participants each had vascular dementia, Lewy bodies dementia, mixed dementia, or frontotemporal dementia. Two were unsure of their dementia type. The mean age was 74 (range 63–83). The mean interview duration for people with dementia was 35 minutes (range 20–52 minutes) for the first interview and 39 minutes (range 31–53 minutes) for the second interview.

### Qualitative findings

Below we discuss how participants engaged with and used MLT, present our thematic analysis of the effects that using MLT had on participants, and identify the mechanisms underlying the observed outcomes. Themes reflecting the outcomes for people engaging with MLT were: a different focus, noticing and reassurance, problem-solving, and reciprocal benefits. The mechanisms that together explain the extent to which MLT was useful for a given individual were support, attitude, and capability.

#### How MLT was used

**Figure 1.**
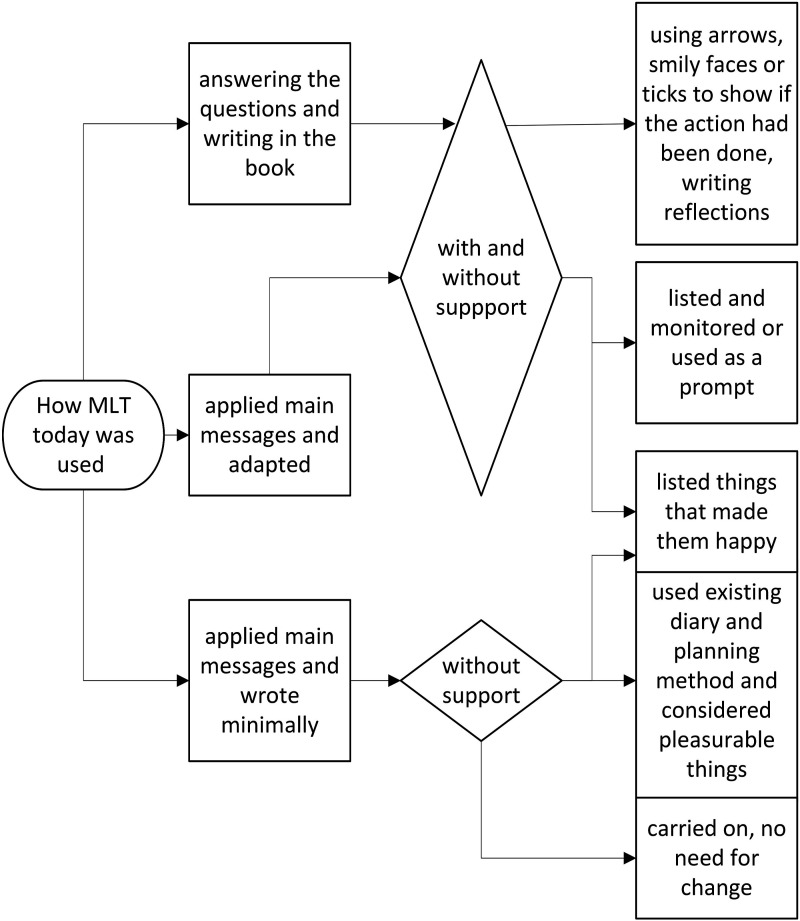
How MLT was used.

A summary of how MLT was used and the observed outcomes is provided in Figure 1.

The frequency of filling in MLT varied; five completed it daily and nine completed it a few days each week, while two used it for one or two days and then stopped, but all attempted to write something to answer the questions. All started by using MLT as intended by answering questions and writing. Seven people identified what they thought were the main messages and used it in another way or tried to carry the principles through to their everyday lives.

##### Answering questions and writing

Although 13 people said they had no problem understanding the questions, only three were able to give examples about identifying actions or changes (04, 05, 09). There was also minimal engagement with the notes area of MLT. This may have been impacted by memory as it would have needed the person to self-monitor and self-reflect, but the instructions on using the notes could have been more explicit in the booklet. The video demonstrated using the notes area to reflect progress, but only one person recalled seeing the video (16). However, eight people mentioned that after completing MLT for a few days, they found it useful to have a comprehensive list of the things that they liked to do. Four marked the things they had listed with a tick or smiley face or date to indicate when these had been done.

##### Applying main messages and writing minimally

Nine people struggled to write in the MLT booklet due to difficulties such as a tremor (02), issues with eyesight or reading (03, 05, 15, 18) or needing help to recall and write things (02, 06, 13, 21SP). Four people thought that completing MLT was time-consuming or unnecessary:

“But it's a bit too detailed, and it's very difficult to sit down all the time filling this in, you know… And my husband, who is my memory at the moment I don't need to plan so much because he's got the memory...” (13)

##### Applying main messages and adapting

All but one identified that MLT reinforced activities they were already doing:

“I have, you know, my… my three things that I try to keep going, one… one is social, you know, one is mental and one is physical. And so, they’re always in the back of my head. I’m totally in support of the book because I… that’s what I already do.” (03)

Twelve people were using other methods to plan or monitor activities such as using an app (07, 15), a computer spreadsheet (02, 05), or some form of diary (01, 04, 07, 13, 15, 16, 18, 21). In three cases, the person with dementia applied what they interpreted as the main principle of MLT and incorporated it into a format that worked for them (02SP, 03, 09). For example, one participant used MLT for ideas and designed a spreadsheet to help list, plan, and monitor all the things that he wanted to do:

“So… but that… that’s all come from your booklet, so although I’m not using the booklet, I’m using the ideas.” (09)

Two indicated that using MLT prompted reflection:

“It just gives me a bit more focus… the main issue there, which was family relationships…. So rather than, you know, following the… the structure in the… in the booklet…I’ve been doing that kind of reflection.” (17)

#### Themes reflecting outcomes of using MLT

The outcomes of using MLT varied in type and level of impact, but ten reported MLT was helpful. Nine participants spoke about how MLT made them think differently about aspects of their lives in a more positive way. People’s feelings about their capabilities seemed to be most relevant to their willingness to engage with and in turn, benefit from MLT. We identified themes that capture the main outcomes experienced by participants. These were: a different focus, noticing and reassurance, problem-solving, and reciprocal benefits.

##### A different focus

Eight people spoke about how working on MLT helped people to think more positively and proactively about how they were using their time:

“It tends to be the practicalities of living…most of the time…that might be something that… that I can build on…spend time planning interesting things or enjoyable things.” (18)

Another six saw that using MLT had made them think differently:

“to actually stop concentrating on the things that you're not happy about. And that is a useful lesson.” (15)

##### Noticing and reassurance

Six people said that at the start they could not think of many things that they liked to do but after using MLT they noticed more of the positive things they were doing:

“A bit down when I do this, because I've been doing the same thing every day, but then I got used to it…I liked writing them down, yeah. And I think it did help me a little bit, [name of researcher], because I could look back, like the last week. ‘Oh, that's where, what did I do last week.' And, you know, things like that.” (10)

Struggling to remember the recent past could affect how people saw themselves, but over time MLT served as a prompt to help them recall what they were doing or generate a ‘list’ of things to do another day. This could result in those things happening more frequently and gave options for daily plans:

“Yeah. It is a prompt. I would say it was a prompt…to think 'What am I going to write tomorrow? Yeah. You know, I never had to think that before, now having this, it makes me think.” (13)

Thinking about the things that made them happy or feel good could help them reconnect with themselves and their identity:

“…if you don’t remember it you don’t remember that you enjoy it…By writing them down, you… then it jogs your memory, that oh yes, I enjoy that.” (09)

“It does help me. It’s helped me in understanding myself as well as other people.” (04)

It was useful for participants to recognise that they were more capable than they thought they might be. This is important because in usual circumstances some people did not realise how much they had done:

“unless you have occasion to look back and review it, you... you tend to lose track of the things that you have got sorted.” (15)

A small number of people spoke about feeling unhappy and disappointed when they noticed they were doing less (10, 11, 16). This could lead to a reduction in using MLT:

“I don't want to... I sort of think too much on the things that make me sad, because it's going to get worse… The only thing is there's loads of things that I can't do, then that would make me sad because I've thought of something I'd like to do, but I can't do it.” (16)

##### Problem-solving

Three people spoke about how recalling the things they were doing also highlighted things that could be done differently. Participants 04, 05, and 09 spoke specifically about using MLT to think more about overcoming difficulties which, if tackled, should in turn lead to fewer concerns and empowerment:

“The thing was I realised that I wasn't doing very much, I'm going to say normal, happy, everyday things, you know? It made me think and be aware and then write down, 'Okay, here's a solution to that. Try that out. And then did it work? Yes, it did.” (05)

##### Reciprocal benefits

The interviews with supporters revealed that there were some benefits for the dyad. Participant 21 received support from his wife to work through the questions in MLT and resolve why he had stopped watching his favourite football TV programme. They identified he was struggling to use the TV controller, and a simple solution was identified so they could both watch what they wanted. This may not have been identified without working through MLT.

In completing the document together, named activities could be collaboratively planned for future days: “So, there… there are a thousand and one things that occupy time from… that potentially get in the way of doing the things that you… your logical mind know are good for oneself, as a carer, or for my wife ...to moving to, ‘Let’s make absolutely sure we do it this week.” (02SP)

Working together on MLT prompted discussions that may not have already been happening:

“We hadn’t consciously for some time sat and said actually, what makes you happy and chatted about, you know… I suppose it helps us because it made us stop and actually say, well, this makes me happy, why don’t I do it, and look for the reasons as to why he’s not doing things.” (21SP)

One of the nominated supporters was a care worker who had other clients with dementia. She could see the benefits of MLT in helping to address low motivation:

“…people with dementia because very... a lot of... they have a lot of apathy. And when they are in that situation, they need to have the strategy there ready to hold onto it and get out of that situation. So, that is... regarding this, the leaflet [MLT booklet] is really useful because they can go back...And they can remind themselves.” (05SP)

### Mechanisms influencing the usefulness of MLT

**Figure 2.**
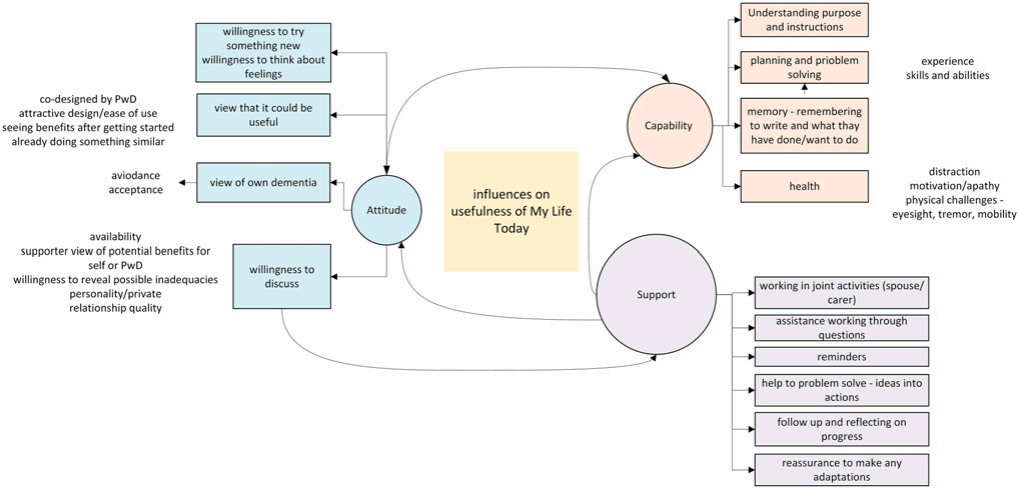
Influences on usefulness of MLT.

Whether people used MLT was influenced by some key mechanisms: support, attitude, and capability. A diagrammatic representation of the relationship between these three mechanisms can be found in Figure 2.

The diagram shows a reciprocal relationship between attitude and capability. Views of capability can influence the need or desire to receive support. Support can have a direct impact on practical assistance to overcome capability issues and can help influence attitude and beliefs about capability.

#### Support

The encouragement provided by researchers seemed to be important for starting to use the document and for continuing; even if the person lived with someone who was supportive, the interactions with the researcher seemed to be useful:

“And that was the most important thing, it feels as if somebody was interested…

You know, I’m… I’m alone with… I’m with (name of partner), I have a wonderful family around me, but it’s not the same.” (04)

Researchers offered encouragement and reassured participants that there were no right or wrong answers. The instructions and the purpose were revisited as needed, and areas of MLT were reiterated. The opportunity for supportive interactions should therefore be recognised as an important part of the MLT ‘package’. People enjoyed the opportunity to talk about some of the things that they had written and were willing to think further about things discussed.

Nine of the participants said they did not discuss MLT with anyone. Three who lived with their spouse said it was private (07, 09, 17), and six of the seven who lived alone said they did not discuss it with anyone. Others (13, 15, 17, 18) said they did not receive support with using MLT but discussed planned events with their spouse:

“She knows I've been doing it, yes. We haven't probably spent a long time discussing it. ...I find it difficult to be sure. I think so. I think... I think it was really... it was thought to be to help me organise my thoughts.”(15)

#### Attitude

Views about the usefulness of MLT were influenced by participants’ experience of making plans or reflecting, and attitudes about whether there were things that could be managed or whether things were out of their control. Two people said they did not like to be reminded of the things they were not doing or struggling with.

Two cases demonstrate differences in attitude and attempts to use the document. Participant 11 considered her usual routine to be repetitive and thought using MLT would emphasise things that made her sad, in particular feeling her dementia was getting worse and the implications for the future. She said:

“...I don't want to... Because I've got Alzheimer's, I know that I shall eventually get to the stage where nobody... I don't know anybody, and I don't want to think about that. (11)

In contrast, participant 04 was used to reflecting, he wrote poetry, and despite several co-morbidities had a positive outlook and contributed to a patient involvement group. He had assistance from his wife to use MLT regularly and was willing to find a way that it could be useful to him:

“I think the way it come over to me, it… it got me thinking… you know you’ve got dementia, but it ticked over in my mind, yeah, this is good, it’s helping me reminisce with things and be a part of something.”

#### Capability

People’s capabilities to work on MLT clearly had an impact on how they used it and whether they found it useful. As well as physical challenges with writing, four people spoke about not being able to answer the planning questions indicating difficulties with reflecting, problem-solving, or identifying specific actions to carry out. Some responses to the question ‘What action could you take to make that thing happen?’ were:

“But I'm not sure what I can do to...to do things any better, if you know what I mean. (07)

and “I can’t say I look back on it, I live each day as it comes, really. Well, it just seems I could make it happen.” (13).

Some people used MLT more like a diary.

“I just wrote mainly the things that I am doing, yeah, that I can do.” (16)

Participant 13 preferred not to answer the questions in MLT but instead discussed the things she liked with her husband:

“I find it hard to sort of get up and think about the day, let alone thinking back on the day, if you know what I mean, to fill in this, because I only get a general feeling of that day…I keep a diary, which is very useful. So, I probably wouldn't use this.”(13)

Only two people said they forgot to fill in MLT (10, 15) but others said they dealt with this by placing the booklet somewhere that would help them to remember (06, 16, 20) or were reminded by someone to fill it in (05). Difficulties recalling things that were enjoyed were overcome by completing MLT closer to the time nice events occurred (07, 09, 10).

In contrast, five people thought it was not necessary to continue to use MLT as they were already doing the things they enjoyed; however, they identified MLT could be useful for other people whose dementia was more advanced (01, 03, 07). Interestingly participant 09 thought it would be suitable for people in the earlier stages of dementia, participant 01 thought that it would be useful for people who are less aware of their behaviour, and participant 13 thought it would be useful for people who do not have help.

## Discussion

This qualitative study based on realist principles investigated the usefulness of MLT, a simple and novel co-produced self-management tool to help people with dementia to monitor and plan meaningful everyday activities that support their well-being. Using a realist approach we have shown why and how MLT can be useful to people with dementia. We have demonstrated that people with dementia can use MLT with minimal support. Participants worked with MLT in the way that suited them, within their capabilities and self-management skills for problem-solving, decision-making, and taking action. The large number of possible combinations of types and degrees of support, attitudes, and capabilities in the relatively small number of people in this study indicates the importance of a flexible and personalised approach to how self-management tools are offered.

People with dementia adapted their use of the booklet by listing the things they were doing that they wanted to continue to do, but rarely made plans. The booklet was kept visible so items they had identified could be picked on any given day. This adaptation is similar to the approach offered by the Pleasant Events Schedule AD (Teri & Logsdon, 1991) designed for use by carers and practitioners, but MLT differed in that it asked people with dementia to identify the things that were important to them. It may be enough for some people with dementia to be encouraged to notice these things in their lives without recording them, but it seems important to check whether the identified things are happening, and if not whether adjustments need to be made.

Recollection and core problem-solving skills were required to fully complete MLT. The five core self-management skills for managing chronic disease identified by Lorig and Holman are: problem-solving, decision-making, resource utilisation, forming a patient/health provider partnership, and taking action (Lorig & Holman, 2003). Willingness to use MLT may have been impacted if these core skills were impeded, making completing MLT difficult, especially when support was not available. Basic problem-solving can be affected in people with mild-to-moderate Alzheimer’s Disease (Passini et al., 1995). In addition, planning actions and setting goals require an element of self-monitoring, drawing on episodic memory to learn, store, and retrieve information, and so can be harder for people with dementia (Banks & Weintraub, 2008). Group self-management interventions aiming to initiate behaviour change for people with mild-to-moderate dementia typically involve a trained healthcare provider and sometimes a carer to assist and guide the person with dementia to set realistic goals and monitor progress (Clare et al., 2019; Farrand et al., 2017; Martin et al., 2015; Quinn et al. 2016a, 2016b). In our study most people with dementia were able to use MLT without a nominated ‘supporter’ but all participants were keen to discuss their responses with researchers who provided a level of support via follow-up, encouragement, reassurance, and guidance.

The mechanisms influencing the usefulness of MLT were support, attitude, and capability. This can be compared to the COM-B theory of behaviour (West & Mitchie, 2020) where, for an intervention to be effective, three factors need to be present: opportunity motivation, and capability. The model proposes that the greater the opportunity (support) and capability the more likely it is the behaviour will occur when the motivation (positive attitude) is present (West & Mitchie, 2020). Motivation is influenced by evaluations of needs and wants, and whether the behaviour is useful or not. The principle behind MLT generally fits with existing attitudes and beliefs about seeking to do rewarding activities. Motivation could be strong when using it was seen as beneficial i.e. it helped to remind them of the things they could do, as opposed to the difficulties associated with dementia. As per the COM-B model, evaluations informing motivation can improve because capability, motivation and opportunities are influenced by feedback loops from performing behaviours (West & Mitchie, 2020); this was seen in our participants who changed their use of MLT to suit themselves based on their experience of using it.

For some, their views about dementia in relation to themselves influenced their evaluation of whether MLT could be useful and their motivation. This supports the common-sense model of self-regulation (Leventhal et al., 2016) where a person’s perception of illness-related threat is derived from cognitive and emotional evaluations of whether and how they can respond. It is also important to acknowledge that how people are adjusting to dementia and how they are managing psychologically will influence their willingness to engage with tools like My Life Today. As indicated by our findings and other studies, people with dementia understand their engagement in activities is important to their sense of self (Caddell & Clare, 2011; Stapley et al., 2023) but for those who do not feel empowered to engage in self-management discussions, or who are influenced by negative beliefs about managing dementia, offering information or other types of support may be appropriate to help increase knowledge and perceived threat and reduce feelings of limited control (Nguyen & Li, 2020) before offering MLT.

Although MLT was intended to encourage people to continue to do things they were already doing it was also intended to encourage people to notice the things they wanted to do more often or restart things they had stopped. However, participants did not always complete the planning questions in MLT as intended but responded in other ways that were useful to them. By allowing flexibility researchers reduced pressure to perform ‘correctly’ and helped to avoid a sense of failure. A flexible approach to intervention delivery is different from other dominant models of self-management using goal-setting approaches such as the widely endorsed CDSMP (Chronic Disease Self-management) (Lorig et al., 1999) based on the social cognitive theory of behaviour change (Bandura, 1986). Central to social cognitive theory is increasing self-efficacy. Improving self-efficacy can help people feel more confident to perform certain behaviours and be achieved successfully in people with early-stage dementia by providing information, goal-setting, and encouraging learning from other people in the group (Quinn et al. 2016b) and is one of the most common objectives in group self-management interventions for people with dementia or mild cognitive impairment (Quinn et al. 2016a). In a self-management course designed by people with dementia (Martin et al., 2015), usual goal-setting stages were simplified to state the goal, when it might be done, and with whom. Like the findings of the current study, the goal and reward were not separate; the goal was to do the identified activity, and the reward was the sense of achievement and pleasure from doing it (Martin et al., 2015). When using MLT, participants noticed the things that they were already doing that were within their capabilities. Some participants naturally self-monitored by seeing the spaces in the MLT booklet being filled, and the things they marked they had done, an important element of ‘noticing’. This may have increased their self-efficacy and may have helped to reinforce a positive self-identity by drawing attention to their capabilities and their daily achievements. Self-monitoring has rarely been included in self-management interventions for people with dementia and is considered more suited for people in the early stages of dementia (Quinn et al. 2016b) but allowing flexibility based on personal capability and offering simplifications and adaptations to usual self-management approaches is likely to be beneficial and should be considered in future studies.

Relatively few people discussed MLT with others or asked for support, but the researcher provided an opportunity for follow-up and to discuss progress. There was evidence of some wishing to keep their thoughts private; avoidance linked to internalised stigma may have protected their self-esteem (Sabat & Harré, 1992) and could be linked to a reluctance to disclose aspects of themselves that are affected by dementia to their spouse to avoid burdening them (Scott, 2022). It could also be a way to maintain control of their own self-management strategies rather than rely on others to be their guide (Dixon et al., 2021). In our study people who did discuss MLT with others were not necessarily those who needed most support. Support occurred when the person with dementia had a close relationship with the person supporting them, and issues were already discussed. Given that people with dementia seemed to benefit from having input from close others or the researcher indicates that some support for using self-management tools could benefit the person with dementia. Other more intensive self-management interventions for people with dementia include social support as a key element (Quinn et al. 2016a).

MLT could be useful for people with dementia who are able to use it by themselves or with support. It would be suitable to offer as a component of a post-diagnostic support package where managing life with dementia is addressed and the person with dementia is willing to consider self-management. Health or care workers could use the tool to stimulate conversations with people with dementia about views about current activities, help complete MLT, and provide an opportunity to discuss progress at a future date. Offering MLT to people with dementia may also assist practitioners in having positive conversations about living with dementia.

Based on the findings of the study there are several adjustments to My Life Today that could increase its usefulness and make it accessible to more people with dementia. These include clearer encouragement to involve someone else to help complete it, space to write a list of meaningful activities, simplified wording of questions to focus on choosing items from the list, and clearer instructions about making simple plans and reflecting on actions. The updated version based on the findings of this study can be found on the IDEAL website. A future study could investigate when would be the best time to offer My Life Today and to whom in the context of the complex variety of different reactions to dementia and different degrees of psychological adjustment at different stages of the dementia journey.

### Limitations

Some participants may not have been able to recall accurately what they had written, or the frequency with which they had completed MLT. Also, while we did not intend to check whether plans were carried out, we hoped this would be captured when discussing what had been written, but the information was not always forthcoming, and we did not collect completed booklets. Although some people with dementia indicated when they had done the things they had written in their booklet, clearer instructions about recording progress and reflections on outcomes could be improved upon. The number of second interviews may have been higher if participants were offered a general follow-up rather than a follow-up aiming to check the use of MLT. There was limited diversity in the small sample so we cannot comment on usefulness for minority groups.

## Conclusion

This study provides a unique insight into the use and benefits of a simple co-produced self-management tool to help people with dementia identify, monitor, and plan the day-to-day things in their lives that are important to them. It has been demonstrated that people with dementia are motivated to maintain their preferred activities and can self-monitor but may benefit from additional support to make changes. Attitudes towards dementia and beliefs about their own capabilities can influence engagement in self-management so exploring this and providing information may be useful when offering such tools. As there is variety in the types and degree of cognitive and physical capabilities, availability and willingness to engage with support, and attitudes towards self-management, a personalised and flexible approach to when and how to offer self-management tools for people with dementia is recommended.

## Supplemental Material

Supplemental Material - Qualitative evaluation of my life today - A co-produced personal tool from the IDEAL programme to help people with dementia monitor valued aspects of their lives Supplemental Material for Qualitative evaluation of my life today - A co-produced personal tool from the IDEAL programme to help people with dementia monitor valued aspects of their lives by Claire Pentecost, Anna Hunt, Rachael Litherland, Catherine Quinn, Catherine Charlwood, Robin G Morris, Linda Clare, in Dementia
