# Supplementary material for: Qualitative evaluation of My Life Today - A co-produced personal tool from the IDEAL programme to help people with dementia monitor valued aspects of their lives
Source: Dementia (London). 2024 Dec 12;24(8):1478–97. doi: 10.1177/14713012241306506 (PMC12508498; doi:10.1177/14713012241306506)
Supplement: Supplemental Material - Qualitative evaluation of my life today - A co-produced personal tool from the IDEAL programme to help people with dementia monitor valued aspects of their lives [file sj-pdf-1-dem-10.1177_14713012241306506.pdf]

IDEAL-2 My Life Today topic guide

Person with dementia 1st interview one month after being asked to start using My Life Today.

For those who have used My Life Today, explore the circumstances surrounding being able to fill it in, and their motivation for filling it in. If it was useful, did they fill it in as we intended and did they identify things to say in each column, or did they use the form slightly differently or write something else that was useful.

For those who say it was not used, not understood or not useful, prompt to understand why or how this was the case.

Opening question – The purpose of My Life Today

- What do you think of My Life Today?
- Did you think it would be helpful when you started? (if yes) [In what way?] (if no) [why not]
    - [What did you think of the instructions and video?]

Circumstances influencing filling in My life Today – context, motivation, assistance, ease of use, difficulties

- Have you used My Life Today? (if no) [why not] (if yes):
    - Did you use it once or more than once?
        - [if more than once] Did you try to use it regularly? [if so, how often i.e. once a week or more? Do you think you will continue to use it?]
    - Were there any difficulties filling it in regularly? [did you set reminders/use strategies to remind you, did anyone help]
- Can you give an example of something you identified that made you happy or feel good? [can you give another example?]
    - Was there anything that was useful about identifying things that help you to feel good and writing these things down? [noticing things, focussing on nice things, telling other people, seeing patterns in types of things, something else?]
    - (if not) why not? [Were the instructions clear? Were you trying to think of small or big things?]
- Did you use the other columns? [That ask how often, how satisfied, what would help, what action could I take] (if no) [why not] (if yes):
    - Was that helpful? Can you give an example? [noticing when things were happening or not, recognising the things you wanted to change, thinking about making them happen more/continue to happen, thinking about planning ahead, making plans]
    - Did you have any issues in thinking of what to write?

Usefulness

(If not covered above) Overall, do you think My Life Today was useful for you? (If no) why? (If yes) how/why?

Involving others External help or support, telling other people

- Did anyone help you in any way? [your husband/wife, or other family member, or a friend, or someone else] (if yes)
    - How did they help you? [what did they do?]

- Did you show it to anyone else and talk about what you had written? (if yes)
    - Can you give an example? [GP or other medical person, anyone else that supports you, someone you wanted to talk to about what you had written]

Aspects of design and content (if not already covered)

- Is there anything that we could improve with the written document or the video? [design colour, content/wording, length, space to write]
- Would you recommend My Life Today to other people [who? Why is that?]

Person with dementia follow up interview

The way people use my Life Today may change over time, there may be different ways they use the information, and different types of things identified. Some may fill it in regularly once a week or more, others may use it intermittently and/or for a particular reason. We want to find out whether the types of things identified change, whether it is used differently over time, and whether My Life Today is still useful after a few months. If it was useful at the start and less useful later, how could it be adjusted to be useful for longer? The follow up will also ask questions based on responses provided in the first interview.

Opening question

- What do you think of My Life Today?

Circumstances influencing filling in My life Today

- Did you use it once or more than once since we spoke [give date]? (If no) why?
  - (if yes) Did you try to use it once a week or more? How has that been going? [strategies to remember, preferred frequency, did anyone help?]
- Did you look at things you had written down before? (If no) why?
  - (if yes) was that useful
  - (if yes) Are the things you have been writing different? Can you give an example? [mention each column and talk about each
- Did you make changes in what you are doing based on the things you wrote? Can you give an example? (If no) why?
  - (if yes) Was that helpful? [Noticing when things were happening or not, rating how satisfied you were, recognising the things you want to change, thinking about making them happen more/continue to happen, thinking about planning ahead, making plans]
- Was My Life Today useful for you since we last spoke to you? (If no) why?
  - (If yes) In what way? Do you think you will continue to use it? why?

Involving others External help or support, telling other people

- Did anyone help you in any way? [same as before or different people] (If no) why?
  - How did they help you? [what did they do?]
- Did you show it to anyone else and talk about what you had written? (If no) why?
  - (if yes) Can you give an example? [GP or other medical person, anyone else that supports you, someone you wanted to show what you had written, same or different people mentioned before]

Aspects of design and content (if not already covered)

- Is there anything else [mention previous responses] that we could improve with the written document or the video?

Nominated friend or family member 1st interview (after the person with dementia has had their interview)

It is possible that the people with dementia may discuss My Life Today with someone close to them. We want to find out whether this was to gain help and support, and/or to share their thoughts or to help with planning for the future.

- Did [name] show you My Life Today?
- Did [name] talk to you about My life Today
    - What did they say?
- At the start did you think My Life Today could be useful? (If yes, how, if not, why not)
- Did you help [name] to use My Life Today in any way?
    - How did you help? [When – what circumstances, what did you do?]
- What did you think of My Life Today?
    - Was it useful for [name]? (If so how, if not, why not)
    - Did you find it useful? (If yes, how? If not, why not)
- Were there any difficulties [name] had with using it?
- Can you suggest any things about My Life Today we can improve on? [design and content of the document and the video]

Nominated friend or family member follow up interview (after the person with dementia has had their follow up interview)

The follow up will ask about things that were said in the last interview as prompts.

- Did [name] show you My Life Today since we last spoke on [give date]?
- Did [name] talk to you about My life Today in that time?
- Did you help [name] to use My Life Today in any way? Has that changed?
    - How did you help? [When – what circumstances, what did you do? Was anything different]
- What do you think of My Life Today now?
    - Was it useful for [name] since [date of last interview]? (If so how, if not, why not)
    - Did you find it useful? (If yes, how? If not, why not)
- Were there any difficulties [name] had using it since [date]?
- Is there anything else about My Life Today we can improve on? [design, content, how it is promoted]
